# Current treatment and outcomes of pediatric gastrointestinal stromal tumors (GIST): a systematic review of published studies

**DOI:** 10.1007/s00383-021-04931-0

**Published:** 2021-06-03

**Authors:** Arimatias Raitio, Adeline Salim, Dhanya Mullassery, Paul D. Losty

**Affiliations:** 1 Department of Paediatric Surgery, Alder Hey Children’s Hospital NHS Foundation Trust, University of Liverpool, Eaton Road, Liverpool, L12 2AP UK; 2 Department of Paediatric Surgery, University of Turku and Turku University Hospital, Kiinamyllynkatu 4-8, 20521 Turku, Finland; 3 Faculty of Health and Life Sciences, University of Liverpool, Liverpool, L69 7TX UK

**Keywords:** Gastrointestinal stromal tumor, GIST, Pediatric, Outcome, Survival

## Abstract

Gastrointestinal stromal tumor (GIST) is a rare cancer of mesenchymal origin mostly seen in adult and elderly populations. Therefore, the prognostic and therapeutic features of pediatric GIST are not clearly defined. Clinical knowledge has been largely extrapolated from case series and adult studies. In this systematic review, we aimed to analyze the health outcome metrics of pediatric GIST. Medline and Embase databases were searched using relevant key terms. The original search retrieved 1,892 titles; 27 studies with 184 patients (68% female) were included for final review. The primary tumors were located in the stomach (165/184, 90%), small bowel (12/184, 7%), and elsewhere (7/184, 4%). Individual patient data were available in 125 cases with a median follow-up of 6.7 years. All patients underwent surgical resection, which varied from wide local excision to total gastrectomy. There were 12 deaths (10%), 65 (52%) patients were alive with no evidence of disease, and 31 cases (25%) were alive with disease. Tumor size > 5 cm, high mitotic index, and spindle morphology were predictive of mortality. Pediatric GIST has a more favorable prognosis and different characteristics versus adult tumors. There is a crucial need for international consensus and specific pediatric guidelines for the treatment of this rare tumor.

## Introduction

Gastrointestinal stromal tumor (GIST) is a cancer of mesenchymal origin arising from the gastrointestinal tract. Overall, one-fifth of soft-tissue sarcomas are GISTs [1], however, the incidence of GIST in the pediatric population is low (about 0.4% of all GIST cases) [2]. The United States Armed Forces Institute of Pathology review reported in a 26-year study only 44 (2.3%) index cases of GIST in pediatric/young adults vs. 1833 GIST in adults [3].

Evidence regarding the clinical behavior of pediatric GIST is limited to extrapolation from adult published case series. However, with advances in genetic sequencing, there is accumulating data now suggesting that pediatric GIST is very different from its adult disease counterpart [4].

This systematic review study aims to critically analyze published clinical outcome(s) on pediatric GIST to identify characteristics (if any) which confer survival advantage and draw comparison(s) to the adult literature.

## Methods

This systematic review was prepared in accordance with Preferred Reporting Items for Systematic Reviews and Meta-Analyses (PRISMA) guidelines [5].

### Search strategy

MEDLINE and Embase databases were searched using the keywords: ‘paediatric’ or ‘pediatric’ or ‘child’ or ‘children’ AND ‘GIST’ or ‘gastrointestinal stromal tumour’ or ‘gastrointestinal stromal tumor’. No time limit was applied, and non-English articles were included due to a low number of relevant publications. Unpublished abstracts from relevant conference proceedings were included in the original search and if deemed relevant, included in the analysis. The two primary authors (AR and AS) independently performed the search and any disagreement was resolved by consensus with the senior author (PDL). The last electronic search was performed on February 28th 2021.

Studies were excluded if they involved mostly adult populations (> 18 years), not related to GIST, or did not include sufficient treatment and outcome data (i.e., type of surgery, recurrence, and mortality). Case reports were excluded. In instances where duplicate studies were identified (similar authors/institutions), we included the more recent/larger/more inclusive study.

### Data extraction and analysis

Following the application of the above exclusion criteria, full-text versions of identified papers were independently reviewed by two primary authors (AR and AS) with final selection approved by the senior author (PDL). The data on patient characteristics, presenting symptoms, diagnostic modalities, treatment, length of follow-up, and patient outcomes was then extracted from the original studies. Detailed information on tumor characteristics, molecular genetics, site, morphology, and presence of metastases was included where available. Standard chemotherapy was classified as any other chemotherapy than tyrosine kinase inhibitors (TRKI).

### Statistical analysis

Chi-square and Fisher’s exact tests were utilized to analyze categorical variables. A Significance level of *p* ≤ 0.05 (two-tailed) was set. Analyses were performed using JMP Pro, version 15.1.0 for Windows (SAS Institute Inc., Cary, NC, USA).

## Results

**Fig. 1:**
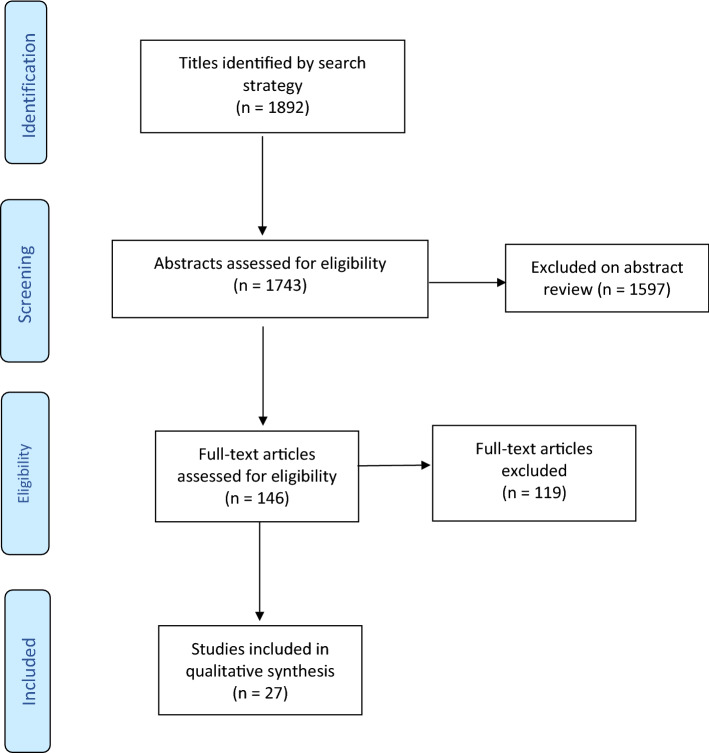
PRISMA study selection flow diagram

The original search through different databases retrieved 1892 articles. A total of 1743 studies were evaluated in the screening of titles and abstracts after duplicates were excluded. Following the application of the exclusion criteria in screening, 146 papers were selected for full-text review. After a full-text review of 146 articles, 27 papers met the eligibility criteria and were selected for the final review (Fig. 1). The published studies covered the time period(s) from 1999 to 2020.

In total, there were 184 patients with pediatric GIST identified which included 126 (68%) females and 58 (32%) males. There was no significant difference in median age at diagnosis between female and male patients: 14 years (range 0–18) and 15 years (range 2–18) respectively (*p* = 0.40). Primary tumors were located in the stomach (165/184, 90%), small bowel (12/184, 7%), and elsewhere (7/184, 4%).

Individual patient data were included in 21 studies with 125 patients. Eleven patients (9%) had associations with Carney’s triad defined as the co-occurrence of GIST, pulmonary chondroma, and paraganglioma [6]. Epithelioid biology was the most common tumor morphology (36/75, 48%) and was more common among female patients (*p* = 0.012, Table 1). The KIT mutation was observed in 5/36 (14%) cases and was more common in males (*p* = 0.029). PDGFRA mutations were observed in only 1/13 (8%) cases.

**Table 1:** Patient demographics

	Median Age (range)	Mortality rate	Morphology (*n* = 75)			Tumor size > 5 cm (*n* = 79)	Wild Type KIT (*n* = 36)	Wild Type PDGFRA (*n* = 13)
			Spindle	Epithelioid	Mixed			
All (*n* = 125)	13 (0–18)	12 (10%)	19 (25%)	36 (48%)	20 (27%)	62 (78%)	31 (86%)	12 (92%)
Males (*n* = 33)	14 (2–18)	3 (9%)	9 (53%)	5 (29%)	3 (18%)	19 (86%)	5 (63%)	1 (100%)
Females (*n* = 92)	13 (0–18)	9 (10%)	10 (17%)	31 (53%)	17 (29%)	43 (75%)	26 (93%)	11 (92%)
*p* value	0.40	0.88	0.012			0.12	0.029	0.76

Surgery was the mainstay of treatment for primary tumors varying from local excision to total gastrectomy. Metastatic disease was reported at the time of primary diagnosis in 12/26 patients (46%). Patients with unresectable primary tumors (13/125, 10%) received standard chemotherapy (3/125, 2%) or TRKI such as imatinib (9/125, 7%) or 2nd generation TRKI (1/125, 1%).

The recurrent disease often presented as local recurrence in 23/56 (41%) and metastatic disease was observed in 35/56 (63%) of these patients. Standard chemotherapy was used for 4/56 (7%), imatinib in 19/56 (34%), and 2nd generation TRKI in 7/56 (13%). Surgery alone was performed in only 11/56 (20%) patients with recurrent GIST.

The most delayed latent GIST recurrence was reported after some 26 years. The median length of follow-up was 6.7 years ranging from 0.25 to 41 years. Complete remission was achieved in 63 (52%) patients while 29 (24%) were alive with the disease. Overall, 12 (10%) deaths were recorded, and 17 patients (14%) were lost to follow-up or no additional data was available.

Age at first diagnosis, gender, and location of tumor were not significantly associated with difference(s) in survival (*p* = 0.38, *p* = 0.88, *p* = 0.31 respectively). There was also no significant difference noted in mortality between patients with and without KIT and PDGFRA mutations (*p* = 0.52). On univariate analysis, spindle morphology, tumor size larger than 5 cm, and high mitotic index were all associated with increased risk of mortality (Table 2).

**Table 2:** Prognostic factors for survival of pediatric GIST

Outcome	Morphology (*n* = 64)			Tumor size (*n* = 79)		Median Mitotic index (*n* = 101) (interquartile range)
	Spindle	Epithelioid	Mixed	< 5 cm	> 5 cm	
Alive	11 (69%)	26 (84%)	17 (100%)	16 (100%)	47 (87%)	6 (3–13)
Dead	5 (31%)	5 (16%)	0	0	7 (13%)	48 (11–65)
*p* value	0.017			0.049		0.003

## Discussion

This systematic review of published studies has shown that GIST is a disease with low mortality in the pediatric population affecting girls more often than boys. Disease recurrence is common despite high overall survival. High mitotic index, tumor size greater than 5 cm, and spindle morphology were all associated with poor patient outcomes.

Before the introduction of TRKI chemotherapy, adult studies reported 5-year survival rates of approximately 50% for GIST% [7, 8]. Since 2000, an improvement in survival among GIST patients has been steadily observed and 5-year survival rates up to 80% have now been reported [2, 9, 10]. Interestingly, over 90% survival in pediatric GIST reported here exceeds that of many adult series. Although short-term follow-up in select cases may be a source of potential bias, our median study length follow-up (6.7 years) was considerably longer than that in the corresponding adult series [7–9].

Tumor size is an identified risk factor for poor prognosis in GIST [7, 11, 12]. Similarly, a higher mitotic index is a predictor of tumor recurrence and/or metastatic disease [13–15]. Concurrent with these findings on adult GIST, our study data demonstrated that tumor size greater than 5 cm and high mitotic index were predictors of mortality also in pediatric GIST. Generally, GIST tumors with a mitotic index higher than 5 are associated with greater risks of adverse outcomes [15]. Interestingly, pediatric GIST appears to have a higher-than-average mitotic activity, as the median mitotic index observed was 6 in those pediatric patients with good outcomes and even higher (48) among non-survivors.

Spindle cell biology predominates in adult GIST followed by mixed spindle-epithelioid or epithelioid morphology [16, 17]. Generally, spindle cell type lesions are associated with low-risk disease, and metastases are more often observed with non-spindle morphology [16]. In pediatric GIST, on the other hand, epithelioid morphology appears most common followed by equal distribution of spindle and mixed tumor morphology(s). Contrary to reports in the adult GIST population, spindle cell morphology was associated with a poorer prognosis in pediatric patients.

Despite equal gender distribution in adult GIST populations, female sex predominated in pediatric GIST, as has been reported previously [4, 18]. Gastric GIST accounts for approximately 50% of all cases encountered in the general population [7, 12, 16] whereas the vast majority of pediatric GIST tumors (> 80%) are located in the stomach as reported here and by Benesch et al. [18]. Previously, male sex [2, 19] and non-gastric location [2] have been identified as adverse prognostic factors in GIST. However, with female preponderance and a higher proportion of gastric tumors observed compared to the adult populations this may explain in part the better outcomes recorded in pediatric patients. Although KIT and PDGFRA genotyping provide important biomarker data for the clinical management of GIST [20], neither here were found to be strongly linked with prognosis in this current study possibly due to the low number of studies reporting these data.

## Limitations

Some limitation(s) of the current systematic review relate to variations in data reporting in the eligible published studies. Hence, individual patient data were not fully available in all studies included in this review. Furthermore, the analyses on tumor genotype and morphology were based on a small subgroup of pediatric patients only. Finally, all included GIST studies analyzed were retrospective cohort populations.

## Conclusions

In conclusion, pediatric GIST carries a better overall prognosis than its adult counterpart. Regardless, very late recurrences do occur highlighting the crucial importance of lifetime after-care follow-up. Tumor size (> 5 cm), high mitotic index, and spindle cell morphology are prognostic for poor outcome(s).

## Data Availability

The data that support the findings of this study are available from the corresponding author upon reasonable request.
